# Radiomics-Based Outcome Prediction in Transarterial Radioembolization (TARE) of Hepatic Malignancies: A Systematic Review and Quality Appraisal

**DOI:** 10.3390/diagnostics16203322

**Published:** 2026-10-13

**Authors:** Richard T. Grainger, Hugo C. Temperley, Omar Abu Saadeh, Niall J. O’Sullivan, Benjamin M. Mac Curtain, Kevin P. Sheahan, Ian Brennan, Zi Qin Ng, Tim Mitchell, Michael E. Kelly, Julian Soares, Hooman Yarmohammadi

**Affiliations:** 1 Royal Darwin Hospital, Tiwi, NT 0810, Australia; 2 Department of Radiology, St James’s Hospital, D08 NHY1 Dublin, Ireland; 3 Trinity St James’s Cancer Institute, Trinity College Dublin, D08 NHY1 Dublin, Ireland; 4 School of Medicine, Royal College of Surgeons in Ireland, D02 YN77 Dublin, Ireland; 5 Northwell Health, New Hyde Park, NY 11040, USA; 6 Department of Urology, Lenox Hill Hospital, New York, NY 10075, USA; 7 Department of Colorectal Surgery, Royal Perth Hospital, Perth, WA 6847, Australia; 8 School of Surgery, University of Western Australia, Perth, WA 6009, Australia; 9 Department of Gastroenterology and Hepatology, Royal Perth Hospital, Perth, WA 6000, Australia; 10 Discipline of Surgery, School of Medicine, Trinity College Dublin, the University of Dublin, D02 PN40 Dublin, Ireland; 11 Department of Interventional Radiology, Royal Perth Hospital, Perth, WA 6000, Australia; 12 Department of Radiology, Memorial Sloan Kettering Cancer Center, 1275 York Avenue, New York, NY 10065, USA

**Keywords:** radiomics, transarterial radioembolization, TARE, Y90, SIRT, hepatocellular carcinoma, liver metastases, treatment response, survival, machine learning

## Abstract

**Background:** Transarterial radioembolization (TARE) with Yttrium-90 (^90^Y) microspheres is an established locoregional treatment for primary and secondary hepatic malignancies. Treatment response is heterogeneous, and accurate pre-procedural prediction of outcome remains a clinical challenge. Radiomics, defined as the high-throughput extraction of quantitative imaging features, may capture subvisual tumour heterogeneity that might be predictive of response, therefore enabling individualised patient selection and treatment planning. **Objectives:** To systematically review the current evidence on radiomics-based models derived from pre- and post-procedural imaging for predicting treatment response and survival outcomes in patients with primary and secondary hepatic lesions that were treated with TARE, and to evaluate the methodological quality of included studies. **Methods:** A systematic literature search was conducted across PubMed, Embase, Scopus, and Web of Science. Studies reporting radiomics, texture analysis, dosiomics, or machine learning applied to imaging in patients with hepatic lesions treated with TARE, with at least one reported performance metric, were included. Methodological quality was assessed using the Radiomics Quality Score (RQS) and risk of bias and applicability using the Prediction model Risk Of Bias ASsessment Tool (PROBAST). Due to substantial heterogeneity, a narrative synthesis was performed. **Results:** Twenty-one studies encompassing 1734 patients were included (up to 73 patients may be counted twice because two cohorts overlap). Studies evaluated patients with hepatocellular carcinoma (HCC; *n* = 12), intrahepatic cholangiocarcinoma (iCC; *n* = 2), colorectal cancer liver metastases (CLMs; *n* = 3), and mixed tumour cohorts (*n* = 4). Imaging modalities included MRI (*n* = 9), CT (*n* = 5), ^18^F-FDG PET or PET/CT (*n* = 3), ^90^Y PET or SPECT (*n* = 2), cone-beam CT (*n* = 1), and CT perfusion (*n* = 1). All studies were retrospective, including two post hoc analyses of randomised trial cohorts, and none used external validation. Eighteen studies reported an area under the curve (AUC). The highest reported AUC ranged from 0.71 to 0.90 in eight studies with no validation, from 0.73 to 1.00 in six studies using internal cross-validation, and from 0.80 to 0.90 in four studies using an internal hold-out set. The median RQS was 2 (interquartile range 1 to 5; maximum 36), and all 21 studies had a high overall risk of bias on PROBAST. **Conclusions:** Radiomic models for predicting TARE outcomes show moderate to high apparent discrimination, but the evidence comes from small retrospective cohorts, is at high risk of bias, and has not been externally validated. Adding clinical variables to radiomic features produced only small gains. Prospective, multi-centre studies with standardised segmentation, harmonised radiomics pipelines, and robust external validation are needed before routine clinical adoption.

## 1. Introduction

Hepatocellular carcinoma (HCC) is the sixth most common cancer worldwide and the third leading cause of cancer-related mortality, with rising incidence historically attributable to chronic viral hepatitis, particularly hepatitis B, alongside alcohol-related liver disease [1,2]; more recently, metabolic dysfunction-associated steatotic liver disease (MASLD) has emerged as the fastest-growing cause of HCC, driving much of the increasing incidence in Western countries, while chronic hepatitis B remains the dominant aetiology in Asia and Africa. Hepatitis C-related HCC, by contrast, has declined markedly following the introduction of direct-acting antivirals. Colorectal cancer liver metastases (CLMs) represent the most common cause of death in patients with colorectal cancer [3], while intrahepatic cholangiocarcinoma (iCC) carries a poor prognosis with limited systemic options [4]. For patients with unresectable disease, transarterial radioembolization (TARE), also known as Selective Internal Radiation Therapy (SIRT) or ^90^Y radioembolization, has emerged as an important locoregional therapy, delivering high-dose internal radiation selectively to hepatic tumours via ^90^Y-labelled microspheres [5,6].

TARE exploits the predominantly arterial blood supply of hepatic tumours relative to the portal-dominant supply of normal hepatic parenchyma, enabling selective tumour irradiation. It is employed as a bridge or downstaging strategy prior to resection or transplantation, in the palliative setting, and increasingly as a curative-intent radiation segmentectomy in selected patients with HCC [7,8]. Despite demonstrated efficacy in improving overall survival and hepatic progression-free survival, treatment response is highly heterogeneous, reflecting the complex interplay of tumour biology, hepatic reserve, dosimetric factors, and procedural variables [9,10].

Current pre-treatment assessment in HCC relies primarily on the Barcelona Clinic Liver Cancer (BCLC) staging system, Child–Pugh score, alpha-foetoprotein (AFP), and ^99m^Tc-macroaggregated albumin (^99m^Tc-MAA) SPECT/CT dosimetric planning. However, these tools demonstrate only modest ability to predict individual treatment response, highlighting an unmet need for more accurate biomarkers [11,12]. Composite scores such as MAAPE (Model for End-stage Liver Disease score, albumin, alpha-foetoprotein, portal vein involvement, and Eastern Cooperative Oncology Group performance status) have also been proposed to predict survival following radioembolization, although this has not been externally validated [13]. These parameters are specific to HCC arising in the context of underlying cirrhosis and are not directly applicable to CLM or iCC.

Radiomics refers to the high-throughput extraction and analysis of large numbers of quantitative imaging features including shape, first-order statistics, and higher-order textural features from standard medical images [14,15]. These features can capture subvisual tumour heterogeneity reflecting underlying biology, including tumour cellularity, vascularity, and necrosis, information invisible to the human eye on qualitative imaging assessment [16,17]. By coupling radiomics with machine learning (ML), multi-feature predictive models can be constructed that may outperform conventional clinical variables.

This review aims to comprehensively evaluate the current evidence on radiomics-based predictive modelling in TARE across all hepatic tumour types and imaging modalities, as an update to the prior systematic review by Mirza-Aghazadeh-Attari et al. which identified 15 eligible studies through September 2023 [18].

## 2. Methods

### 2.1. Study Design and Reporting Guidelines

The methodology employed in this study was similar to that used in our group’s previous radiomics systematic reviews [19,20,21,22], with adaptations to reflect the specific clinical context of TARE. This study is a systematic review conducted in accordance with the Preferred Reporting Items for Systematic Reviews and Meta-Analyses (PRISMA 2020) [23]. The review protocol was registered on PROSPERO (Registration ID: CRD420261381083). The registered protocol specified a meta-analysis, which was not undertaken because of the heterogeneity described in Section 2.6, so the review is reported as a systematic review with narrative synthesis. PROBAST was used in place of the QUADAS-2 tool listed in the protocol because it is designed for studies developing or validating prediction models.

### 2.2. Search Strategy

A systematic literature search was performed across PubMed (via Medline), Embase, Scopus, and Web of Science, supplemented by a search of ClinicalTrials.gov, with no date restrictions applied. The final search was conducted on 28 May 2026. Full search strings are provided in Supplementary Material S1.

### 2.3. Inclusion and Exclusion Criteria

Studies were included if they were: (1) retrospective or prospective cohort studies; (2) enrolling adult patients (≥18 years) with hepatic lesions (primary or secondary) undergoing TARE, ^90^Y, or SIRT; (3) applying radiomics, texture analysis, dosiomics, or ML to any imaging modality [CT, MRI, PET/CT, SPECT/CT, cone-beam CT (CBCT), or combinations]; (4) reporting at least one quantitative performance metric (AUC, sensitivity, specificity, C-index, accuracy); and (5) published in English.

Studies were excluded if they were: reviews, editorials, conference abstracts, or case reports; studies on transarterial chemoembolization only; studies applying dosimetry or dose-volume histogram (DVH) analysis only without explicit radiomic feature extraction from imaging; end-to-end deep learning models without radiomic feature extraction; animal or phantom studies only; or studies reporting no performance metric. Studies representing duplicate patient populations from already-included studies were also excluded.

### 2.4. Study Selection and Data Extraction

All identified records were imported into Covidence and duplicate entries removed. Two reviewers (RG and HT) independently screened titles and abstracts, then full texts, against the inclusion criteria. Disagreements were resolved by a final decision of the senior author (MK). Data extraction was performed independently by two reviewers (RG and HT) using a standardised form capturing: first author, year, journal, tumour type, imaging modality, timing, segmentation method, radiomics software, number of patients, outcome definition, response criteria, model type, performance metrics, and validation strategy. Disagreements were resolved by consensus. No automation tools were used for screening or extraction. Where a value was not reported, it was recorded as not reported and no assumptions were made. Study authors were not contacted for missing data. For every study, all reported AUC, C-index, sensitivity and specificity values were extracted for each model, and the highest reported AUC of a model containing radiomic features was used in the summary.

### 2.5. Methodological Quality Assessment

Methodological quality was assessed using the Radiomics Quality Score (RQS) [15], and risk of bias and applicability using the Prediction model Risk Of Bias ASsessment Tool (PROBAST) [24], which is designed for studies developing or validating prediction models. Each study was scored independently by two reviewers (RG and HT), and disagreements were resolved by a final decision of the senior author (MK). The scoring conventions and the results are presented in Supplementary Materials S2 (RQS) and S3 (PROBAST).

### 2.6. Data Synthesis

Substantial heterogeneity in acquisition parameters, segmentation strategies, radiomics workflows, feature extraction methods, machine learning algorithms, and outcome definitions precluded quantitative meta-analysis. Instead, a structured narrative synthesis was undertaken. Reported performance metrics, including areas under the receiver operating characteristic curve (AUCs) for radiomics-only, clinical-only and combined models, were collated and summarised descriptively. For each study, the highest reported AUC of a model containing radiomic features was tabulated, and AUC ranges were summarised separately by validation strategy (none, internal cross-validation, internal hold-out and external validation), because estimates obtained with different strategies are not comparable. No pooled or median estimates were calculated. Publication bias and the certainty of evidence (for example with GRADE) were not formally assessed because no quantitative synthesis was performed.

## 3. Results

### 3.1. Search Results

**Figure 1:**
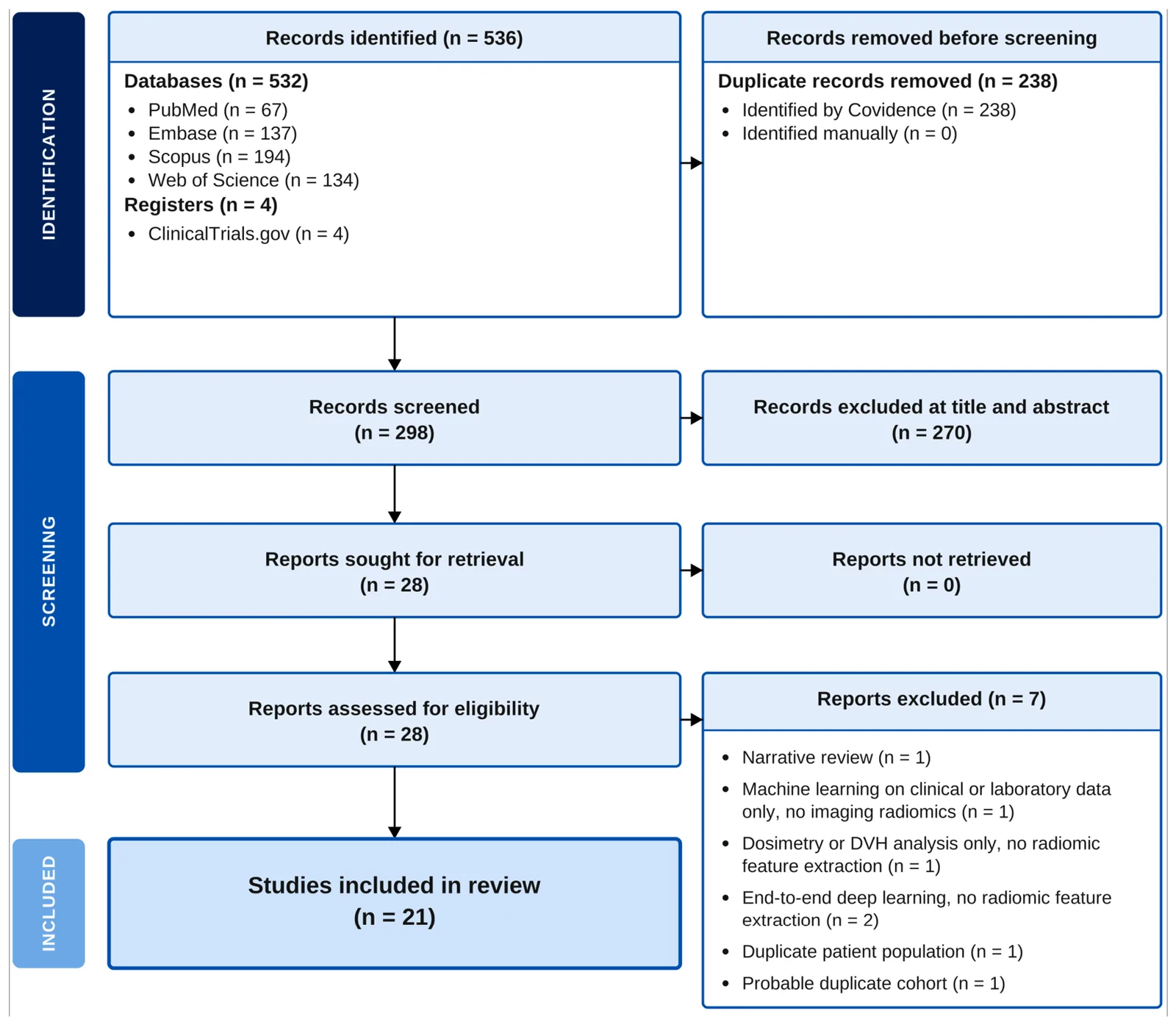
PRISMA 2020 flow diagram.

The systematic literature search yielded 536 records: 532 from the four databases and 4 from ClinicalTrials.gov. Following removal of 238 duplicates, 298 studies were screened by title and abstract. After initial screening, 28 full texts were assessed for eligibility. Seven studies were excluded at full-text review: one narrative review [25], one study applying ML to clinical/laboratory parameters without imaging radiomics [26], one study performing DVH/dosimetry analysis only without radiomic feature extraction [27], two end-to-end deep learning approaches without explicit radiomic feature extraction [28,29], one representing a duplicate patient population [30], and one representing a probable duplicate cohort [31]. Ultimately, 21 studies met the inclusion criteria and were included in the final analysis. A PRISMA flow diagram is presented in Figure 1.

### 3.2. Methodological Characteristics and Quality

All 21 included studies were retrospective in design. Most were single-centre; Mosconi et al. [32] was multi-centre. Two studies were post hoc analyses of prospectively collected randomised trial cohorts: Ocal et al. (SIRFLOX, *n* = 491, of whom 239 received chemotherapy alone) [33] and Saalfeld et al. (SORAMIC sub-study, *n* = 297, of whom 147 received sorafenib alone) [34]. Because about half of the patients in these two cohorts did not receive TARE, they are described as trial-derived cohorts. Publication years ranged from 2012 to 2026. Table 1 summarises the methodological characteristics of all included studies [32,33,34,35,36,37,38,39,40,41,42,43,44,45,46,47,48,49,50,51,52].

Methodological quality was low. The median RQS was 2 of a possible 36 (interquartile range 1 to 5; range −5 to 12), and the highest score (Marinelli et al., 12) was 33% of the maximum. No study used external validation, prospective enrolment, decision-curve or other clinical utility analysis; calibration was assessed in only one study (Wei et al.); a phantom study of feature repeatability was performed in only one study (Wei et al.); and inter-observer segmentation agreement was assessed in six studies. Most cohorts were small (fewer than 100 patients). On PROBAST, the overall risk of bias was high in all 21 studies, and the analysis domain was rated high in every study, chiefly because of few outcome events relative to the number of candidate predictors, feature selection or oversampling before validation, absence of calibration, and lack of external validation. Applicability was a high concern for Ocal et al. and Saalfeld et al. because roughly half of their patients did not receive TARE. Detailed results are provided in Supplementary Materials S2 and S3.

### 3.3. Participant Characteristics

The 21 included studies encompassed 1734 patients as reported (Table 1). Of these, 386 were comparator-arm patients in Ocal et al. and Saalfeld et al. who did not receive TARE, and up to 73 patients in Stocker et al. were also reported in Marinelli et al., so the number of unique TARE-treated patients lies between 1275 and 1348. Individual study populations ranged from 16 to 491 patients. Studies evaluated HCC (*n* = 12 studies), CLM (*n* = 3), iCC (*n* = 2), and mixed tumour cohorts (*n* = 4; Gensure, Kobe, Reimer and Wei). Table 1 summarises participant and study characteristics.

### 3.4. Imaging Modalities and Radiomics Workflow

#### 3.4.1. Imaging Modalities

MRI was the most frequently utilised modality (nine studies), used for pre-procedural tumour characterisation, early post-TARE response assessment, and combined baseline/follow-up analysis. Sequences included contrast-enhanced T1-weighted (CE-T1W), T2-weighted (T2W), T2W with fat suppression, diffusion-weighted imaging (ADC maps), and hepatocellular phase imaging. CT was employed in five studies, encompassing portal venous phase, triphasic and quadriphasic acquisitions, and CT perfusion was used in one further study (Reiner et al.) [35]. ^18^F-FDG PET or PET/CT was used in three studies, and ^90^Y PET/CT or SPECT/CT in two. Cone-beam CT (CBCT), acquired during the angiographic procedure itself, was used in one study [36]. Post-procedural imaging was employed in four studies [37,38,39,40].

#### 3.4.2. Segmentation

Segmentation approaches varied considerably (Table 2). Contouring was manual or reader-defined in ten studies, semi-automated (threshold, region-growing or interactive tools) in nine, automated with a deep learning model (nnU-Net) in one [33], and manual for the tumour with deep learning for the whole liver in one [40]. Inter-observer reproducibility of segmentation was assessed in six studies (Kobe et al. [36], Stocker et al. [41], Marinelli et al. [42], Reiner et al. [35], Ince et al. [45] and Roll et al. [51]); for example, ICC 0.83 in Marinelli et al. The remaining 15 studies did not assess it, which is a significant methodological gap given the well-established sensitivity of radiomic features to segmentation variability.

#### 3.4.3. Feature Extraction, Selection, and Machine Learning

PyRadiomics was the most commonly used feature extraction package (eight studies), followed by LIFEx (five studies) and Olea Sphere (two studies); CGITA, mint Lesion, an in-house MATLAB toolbox, and 3D Slicer without a stated package were each used in one study, and custom code in two (Table 2). The number of candidate features ranged from 7 to 1128, with feature selection performed using LASSO or elastic-net regularisation, Pearson or Spearman correlation filtering, ICC- or CCC-based reproducibility filtering, XGBoost feature importance, Boruta, or univariable testing. Three studies (Mahmoud, Reimer and Reiner) performed no feature reduction and no correction for multiple testing. Final models incorporated between 2 and 20 selected features. ML algorithms included logistic regression, SVM, random forests, XGBoost, LightGBM, MLP ANN, LASSO Cox regression, and radiomic signature approaches. Table 2 summarises imaging parameters and performance metrics.

### 3.5. Outcomes and Predictive Performance

Response was assessed using Response Evaluation Criteria in Solid Tumours version 1.1 (RECIST 1.1), modified RECIST (mRECIST), or European Association for the Study of the Liver (EASL) criteria, depending on tumour type and study design. Eighteen studies reported an AUC: seventeen for treatment response and one (Saalfeld et al.) [34] for 1-year survival (Table 3). Because estimates obtained with different validation strategies are not comparable, they were not pooled, and Table 4 gives the range of the highest reported AUC in each validation stratum. Eight studies reported apparent performance with no validation (range 0.71 to 0.90; this includes Roll et al., whose logistic model was fitted on the same validation set on which the AUC is reported), six used internal cross-validation (range 0.73 to 1.00), and four used an internal hold-out set (range 0.80 to 0.90), with small test sets (about 30 to 50 patients or 21 lesions) and wide confidence intervals where reported. No study used external validation. Three studies [33,43,44] reported no AUC or C-index, and one [35] reported an AUC of 0.81 without a confidence interval. Radiomics-containing models had higher AUCs than clinical-only models in the six studies that reported both [41,42,45,46,47,48], whereas adding clinical or dosimetric variables to radiomics gave small increments (0.01 to 0.04) in four of the five studies that reported both models and no gain in one (Topcuoglu et al.) [49]. No single radiomic feature or feature class emerged consistently across studies.

#### 3.5.1. Treatment Response—MRI-Based Studies

Seven studies evaluated pre-procedural MRI radiomics for treatment response prediction [41,42,45,46,47,48,50]. All seven reported an AUC; the highest reported values ranged from 0.74 to 1.00 (0.74 to 0.94 excluding Aujay et al., *n* = 22, LOOCV), none used external validation, and only Marinelli et al. used a hold-out set. Radiomics-containing models had higher AUCs than clinical-only models in the studies that reported both [41,42,45,46,47,48], but the increment from adding clinical variables to radiomics was small. The largest single-centre MRI study (Stocker et al., *n* = 154) [41] achieved an AUC of 0.736 with radiomics, clinical parameters and AFP, compared with 0.531 for clinical parameters alone, in repeated internal cross-validation, illustrating that performance is more modest in larger cohorts than in smaller studies. Stocker et al. state that 73 patients were also reported in previous studies, including Marinelli et al. [42], so the two results are not independent. Baseline pre-treatment features were most heavily weighted across models [42].

#### 3.5.2. Treatment Response—CT-Based Studies

Five studies evaluated CT-based radiomics [32,33,34,43,51]. Three reported an AUC: 0.896 for Mosconi et al. (apparent performance, no validation), 0.75 for Roll et al. (95% CI 0.48 to 1.00; the logistic model was fitted on the same validation set, so this is an apparent estimate) and 0.803 for Saalfeld et al. (1-year survival). Texture features reflecting intratumoral heterogeneity, including entropy, energy, and grey-level matrix parameters, were the most consistently predictive across tumour types [32,35,36,51], consistent with the biological rationale that heterogeneity governs differential microsphere uptake. High-risk radiomic profiles correlated with significantly shorter overall survival (OS) in CLM (3.4 vs. 6.4 months, *p* < 0.001) [51], suggesting prognostic as well as predictive utility. Peri-procedural CBCT radiomics achieved an AUC of 0.85 in a hold-out set of 21 lesions, split at lesion rather than patient level, without dedicated pre-treatment imaging [36]; this is practically attractive but requires validation in independent patients.

#### 3.5.3. Treatment Response and Survival—PET/CT-Based Studies

Three studies evaluated ^18^F-FDG PET or PET/CT radiomics. Of these three studies, two evaluated HCC [44,52] and one evaluated CLM [49], indicating that the reduced incremental value of clinical features in PET-based models discussed below is not simply attributable to a shift away from HCC, since HCC still comprised the majority of the PET literature. Two reported an AUC: 0.827 for metabolic tumour volume without validation [52] and 0.90 in a 30-patient hold-out set [49]. PET-based studies identified metabolic and textural features as predictors of response and survival, but no PET study was externally validated. In the one PET study that compared them, adding clinical features did not improve on radiomics alone [49] (AUC of 0.88 versus 0.90), so no conclusion can be drawn about differences between modalities. Whole-liver PET radiomics independently predicted progression-free survival (PFS) and OS, suggesting that background liver metabolic texture captures prognostic information beyond the target lesion [44].

#### 3.5.4. Survival Prediction and Body Composition Radiomics

Two studies evaluated radiomics for survival prediction using trial-derived cohorts. Saalfeld et al. reported that CT body composition radiomics predicted 1-year survival in SORAMIC data (*n* = 297), with test-set AUC of 0.803 (95% CI 0.693 to 0.913) in the SIRT plus sorafenib arm and 0.758 (95% CI 0.638 to 0.878) in the sorafenib arm; the confidence intervals overlap widely, so a SIRT-specific radiomic survival signal is not established [34]. Ocal et al., using SIRFLOX data (*n* = 491), identified CT radiomic features interacting with treatment allocation that were associated with overall survival benefit from SIRT, with automated nnU-Net segmentation checked against manual expert segmentation in 80 patients; no AUC or C-index was reported and the signature was not validated [33]. Both studies draw on prospectively collected trial data but are retrospective post hoc analyses, about half of their patients (239 of 491 and 147 of 297) did not receive TARE, and both were rated at high risk of bias on PROBAST (Supplementary Material S3); their findings require prospective external validation [33,34].

#### 3.5.5. Post-Procedural and Dosiomics Studies

All four reported an AUC, with highest reported values of 0.71 (Mahmoud et al., no validation, *p* = 0.07), 0.729 (Wei et al., nested cross-validation), 0.83 (Reimer et al., no validation) and 1.00 (Mansouri et al., nested cross-validation). Results from Mansouri et al. 2025 (AUC of 1.00, *n* = 17) should be interpreted with extreme caution given the very small cohort size and absence of external validation [40]; excluding this study, the highest reported values were 0.71 to 0.83. Post-procedural radiomics nonetheless offers the conceptually distinct advantage of capturing actual treatment effect rather than predicting it from baseline tumour characteristics. In Wei et al., the model combining post-therapy ^90^Y PET radiomics with absorbed dose was not significantly better than either alone (AUC of 0.729 versus 0.713 for each; C-index for progression of 0.803 versus 0.754 for dose alone) [39], so complementarity is suggested but not demonstrated. Post-treatment MRI texture analysis performed within a mean of 2.2 days of TARE identified progressive disease earlier than RECIST 1.1 criteria [37], highlighting a potential role for early post-procedural imaging in treatment monitoring. The analysed time point in that study was chosen using the outcome, which may inflate performance. The clinical utility of post-procedural radiomics is, however, constrained by the requirement for early follow-up imaging that is not universally available in routine practice, and by the small cohort sizes in all four studies (range *n* = 17–37). A summary of performance metrics is presented in Table 3.

**Table 3:** Discriminative performance of models with radiomic features, by study and validation type.

Study	Year	Outcome	Clinical or Dose Model AUC; Sens, Spec	Radiomics Only AUC; Sens, Spec	Clinical + Radiomics AUC; Sens, Spec	Validation of Reported Estimate
Aujay [50]	2022	Response, mRECIST 6 mo	NR	1.00; sens 100%, spec 100% *	NR	LOOCV
Ballı [48]	2024	Response, mRECIST 6 mo	0.769; sens 69%, spec 74%	0.839 (T2W FS); sens 77%, spec 83%	0.880; sens 92%, spec 78%	None
Blanc-Durand [44]	2018	OS and PFS	NR	No AUC; log-rank *p* = 0.007 (OS), *p* = 0.0004 (PFS)	NR	None
Gensure [43]	2012	Serologic response	NR	No AUC; accuracy 96%	NR	LOOCV
Ince [45]	2023	Response, EASL 3 mo	0.82 to 0.83	NR	0.94 (SVM, LR)	Nested 5-fold CV
Kobe [36]	2021	Lesion response, RECIST 1.1	NR	0.85; sens 94.2%, spec 67.7%	NR	Hold-out (21 of 104 lesions)
Mahmoud [38]	2024	Response, mRECIST 9 mo	NR	0.71; sens 93%, spec 62%; *p* = 0.07	NR	None
Mansouri [40]	2025	Response, mRECIST 3 mo	NR	1.00 (^90^Y SPECT); at least 0.91 (MAA) *	1.00 (^90^Y radiomics + clinical) *	Nested 3-fold CV
Marinelli [42]	2023	Complete response, mRECIST	0.58 (imaging criteria 0.59)	0.89; sens 80%, spec 88%	NR	Hold-out (*n* = 30 of 76)
Mosconi [32]	2020	Objective response	NR	0.896 (0.814 to 0.977), apparent	NR	None
Ocal [33]	2026	OS; SIRT benefit	NR	No AUC or C-index; log-rank *p* < 0.001	NR	None
Reimer [37]	2018	Progressive disease	NR	0.83; sens 75%, spec 85% (0.73 to 0.83 across parameters)	NR	None
Reiner [35]	2016	Response, mRECIST	NR	0.81; sens 88%, spec 75%	NR	None
Roll [51]	2024	Response, RECIST 1.1 3 mo	NR	0.75 (0.48 to 1.00); sens 83%, spec 62%	NR	None (model fitted on validation set)
Saalfeld [34]	2023	1-year survival	NR	0.758 (sorafenib); 0.803 (SIRT + sorafenib)	NR	Hold-out (one-third)
Sarioglu [46]	2025	Treatment response	0.65	0.92 (CE-T1W); 0.77 (T2W)	NR	5-fold CV
Karahan Şen [52]	2022	Complete response	NR	0.827 (MTV); sens 78.9%, spec 78.6%	NR	None
Sozutok [47]	2024	Complete response	0.736	0.754 (T1W); 0.850 (T2W)	0.769 (T1W); 0.870 (T2W)	None
Stocker [41]	2025	Response, mRECIST 6 mo	0.531; sens 78.1%, spec 27.9%	0.726; sens 70.1%, spec 65.1%	0.736; sens 70.6%, spec 66.2%	Repeated 80:20 CV (×100)
Topcuoglu [49]	2026	Local response, PERCIST	NR	0.90 (RF; 0.71 to 1.00); sens 100%, spec 67%	0.88 (RF)	Hold-out (*n* = 30 of 74)
Wei [39]	2020	Lesion response	Dose only 0.713	0.713 (0.685 to 0.741)	0.729 (0.702 to 0.758)	Nested CV

AUC = area under the receiver operating characteristic curve; sens = sensitivity; spec = specificity; NR = not reported. Sensitivity and specificity are given after the AUC, following a semicolon, where reported. * Interpret with extreme caution: cohort of 22 patients or fewer with apparent, leave-one-out or nested cross-validated performance. Where several models or sequences are reported, the highest radiomics-only value or the value for the principal model is shown. For Ballı et al., the combined value is combined model 3 (T2W without fat suppression). Ince et al. report clinical-only AUC of 0.82 to 0.83 and combined AUC of 0.94 (SVM and LR), 0.92 (LightGBM) and 0.88 (RF). Mosconi et al. report apparent performance; 10-fold CV was used only to tune the LASSO penalty. The Wei et al. combined model is radiomics plus absorbed dose, and the C-index for progression was 0.803 (0.790 to 0.815).

**Table 4:** Range of highest reported AUC by validation type.

Validation Type	Studies (n)	Range of Highest Reported AUC	Studies
None (apparent performance)	8	0.71 to 0.90	Ballı, Mahmoud, Mosconi, Reimer, Reiner, Roll, Karahan Şen, Sozutok
Internal cross-validation (including nested and leave-one-out)	6	0.73 to 1.00	Aujay, Ince, Mansouri, Sarioglu, Stocker, Wei
Internal hold-out (random split of the same cohort)	4	0.80 to 0.90	Kobe, Marinelli, Saalfeld, Topcuoglu
External validation (independent cohort)	0	Not applicable	None

Eighteen of 21 studies reported an AUC. Blanc-Durand et al., Gensure et al. and Ocal et al. reported no AUC or C-index. The highest reported AUC per study is shown, which favours optimism, and no pooled estimate is given because of clinical and methodological heterogeneity. Cross-validated estimates are not independent validation. The Marinelli et al. hold-out set was a random subset of the same institutional cohort. Roll et al. are placed in the no-validation stratum because their logistic model was fitted on the same validation set on which the AUC is reported.

## 4. Discussion

This systematic review identified 21 studies evaluating the role of radiomics in outcome prediction for TARE, encompassing 1734 patients (up to 73 counted twice) across a broad range of hepatic tumour types, imaging modalities, and clinical contexts. Compared with the prior systematic review by Mirza-Aghazadeh-Attari et al., which identified fifteen eligible studies through September 2023 [18], this update includes nine studies that were not in that review (eight published between 2024 and 2026 and one, Reiner et al. [35], in 2016). Two of the twenty-one studies were post hoc analyses of randomised trial cohorts (Ocal et al., SIRFLOX; Saalfeld et al., SORAMIC) [33,34], although about half of the patients in those cohorts did not receive TARE. Seven studies were excluded at full-text review, most commonly for applying dosimetry or dose-volume histogram analysis without explicit radiomic feature extraction, using end-to-end deep learning without interpretable radiomic features, or representing duplicate patient populations. These exclusions reflect the evolving and inconsistently defined boundaries between radiomics, dosiomics, and deep learning in the interventional oncology literature.

The overarching finding is that radiomic models show moderate to high apparent discrimination but that the evidence is at high risk of bias. Among the 18 studies reporting an AUC, the highest reported values ranged from 0.71 to 0.90 with no validation, 0.73 to 1.00 with internal cross-validation, and 0.80 to 0.90 with an internal hold-out set; no study used external validation. The highest reported AUCs here (0.71 to 1.00) overlap the ranges reported in our group’s earlier radiomics systematic reviews in anal cancer (0.68 to 1.00) [20], ovarian cancer (0.77 to 0.89 in validation sets) [22] and bladder cancer (median 0.75, range 0.70 to 0.84 in validation sets) [21], whereas the sinonasal review reported higher values (median AUC of 0.91 in validation sets) [19]. Those reviews mostly summarised training or validation-set estimates, addressed different outcomes and appraised quality with QUADAS-2, so the comparison is indicative only. Mirza-Aghazadeh-Attari et al. reported pooled sensitivity (83.5%) and specificity (86.7%) and a summary ROC AUC of 0.92 (95% CI 0.89 to 0.94) for response prediction, and a mean RQS of 11.4; because we applied explicit scoring conventions (for example, cross-validation alone scored as no validation; Supplementary Material S2), our median RQS of 2 should not be compared directly with their figure [18]. This should not be interpreted as clinical readiness. The majority of studies were small, retrospective, single-centre analyses without external validation, and AUC inflation by overfitting in small cohorts is well established in the radiomics literature [14,15]. All 21 studies were at high risk of bias on PROBAST.

Radiomics-containing models achieved higher AUCs than clinical-only models in the six studies that reported both [41,42,45,46,47,48], the largest contrast being Stocker et al. (*n* = 154; AUC of 0.736 with radiomics, clinical parameters and AFP versus 0.531 for clinical parameters alone, in repeated internal cross-validation) [41]. By contrast, adding clinical or dosimetric variables to radiomic features gave only small increments (0.01 to 0.04) in four studies [39,41,47,48] and none in one [49]. The clinical comparators were generally ad hoc models rather than established staging systems, so the incremental value of radiomics over standard care remains unproven. Radiomics may capture subvisual heterogeneity reflecting microarchitecture, vascularity and necrosis, while clinical variables provide systemic context, but the included studies did not test this. In the one PET study that compared them, clinical features added nothing to radiomics [49], and because this is a single study, no difference between modalities can be inferred. Examining findings by tumour type reveals important nuances. HCC was the most studied entity (12 of 21 studies), evaluated across MRI, CT, and PET modalities, with response criteria varying between RECIST 1.1, mRECIST, and EASL. Across HCC studies, pre-procedural MRI-based radiomics most consistently predicted response, with baseline pre-treatment features more heavily weighted than post-treatment delta-features in the majority of models [41,42,45], supporting a pre-procedural patient selection paradigm consistent with the clinical imperative to identify non-responders before rather than after the procedure. For intrahepatic cholangiocarcinoma (two studies), both MRI- and CT-based radiomics demonstrated promising performance, with the multi-centre CT study by Mosconi et al. (AUC of 0.896) particularly notable for its ComBat harmonisation approach to inter-centre imaging variability [32], a methodological step that should be standard practice in multi-centre radiomics research. Ballı et al. similarly demonstrated the predictive value of dynamic MRI-based radiomics in iCC (AUC of 0.839 for the best single sequence and 0.880 for the best combined model, *n* = 36, without validation) [48]; both iCC studies were unvalidated, and only one used MRI. For colorectal cancer liver metastases (three studies), trial-derived radiomics data from Ocal et al. (SIRFLOX, *n* = 491) [33] and PET radiomics data from Topcuoglu et al. (*n* = 74) [49] add to the evidence, but neither was externally validated and Ocal et al. reported no discrimination metric.

The two largest studies in this review draw on prospectively collected randomised trial data but are retrospective post hoc analyses. Ocal et al. conducted a post hoc radiomic analysis of the SIRFLOX randomised controlled trial (*n* = 491), identifying CT radiomic features from pre-treatment portal venous phase imaging that interacted with treatment allocation to predict overall survival benefit from SIRT, using automated nnU-Net segmentation validated against manual expert segmentation in 80 patients [33]. This is the first attempt at a radiomics-based predictive biomarker for treatment selection in TARE, a conceptually important distinction from prognostic models, but the signature was not validated and no discrimination metric was reported, so it does not yet establish that SIRT confers a survival advantage in a defined subgroup. Saalfeld et al. conducted a post hoc analysis of the SORAMIC trial (*n* = 297), demonstrating that body composition radiomics derived from skeletal muscle and adipose tissue segmentation predicted 1-year survival with greater accuracy in the SIRT + sorafenib arm (AUC of 0.803) than sorafenib monotherapy (AUC of 0.758; overlapping confidence intervals), which does not establish that treatment context modifies the prognostic value of body composition radiomics [34]. The involvement of body composition as a radiomic predictor aligns with established literature on sarcopenia as a determinant of TARE outcomes, extending this concept into quantitative imaging-based phenotyping. Both studies are post hoc analyses, about half of their patients did not receive TARE, and their findings require prospective external validation.

The breadth of imaging modalities evaluated in this review reflects the clinical heterogeneity of the TARE patient population and the opportunistic nature of radiomics research. MRI dominated, reflecting its superior soft tissue contrast and clinical primacy for HCC characterisation and treatment response assessment. The use of peri-procedural CBCT by Kobe et al. [36] is particularly noteworthy from a translational perspective: radiomic features extracted from CBCT acquired during the TARE procedure itself, without any requirement for dedicated additional imaging, achieved an AUC of 0.85 for response prediction in a hold-out set of 21 lesions split at lesion rather than patient level. If validated prospectively, intra-procedural CBCT radiomics could provide real-time information to guide microsphere dosing without additional patient burden.

Post-procedural radiomics and dosiomics represent a conceptually distinct dimension of this literature. Four studies evaluated post-procedural imaging, with highest reported AUCs of 0.71 to 1.00 (0.71 to 0.83 excluding the Mansouri et al. 2025 result of an AUC of 1.00 in *n* = 17, which should be interpreted with extreme caution [40]). Post-procedural radiomics offers the advantage of capturing the actual biological response to treatment rather than predicting it from pre-treatment characteristics alone. The combination of post-therapy ^90^Y PET radiomics with absorbed dose metrics by Wei et al. was not significantly better than either alone (AUC of 0.729 versus 0.713 for each; C-index for progression of 0.803) [39] so complementarity is suggested but not demonstrated. Reimer et al. reported that post-treatment MRI texture analysis performed within a mean of 2.2 days of TARE could discriminate progressive disease (AUC up to 0.83), although the analysed time point was chosen using the outcome [37]; if confirmed, this could have implications for early treatment monitoring. Interpretation of post-SIRT imaging nonetheless remains inherently complex, given overlapping post-treatment inflammatory and necrotic change; imaging features specifically predictive of local recurrence, as distinct from treatment response in general, would represent a particularly useful clinical application of this approach. In practice, post-treatment dosimetry is already reviewed to guide follow-up, with re-imaging typically performed at three months where tumour uptake and absorbed dose appear adequate; a radiomic signature capable of predicting response in this pre-selected population, who by definition had favourable pre-treatment features and therefore proceeded to treatment, would be of considerable additional clinical value. The dosiomics field in TARE remains nascent, with the only dedicated dosiomics study to date too small to draw firm conclusions (*n* = 17) [40], but the conceptual framework is compelling and warrants dedicated prospective investigation.

Substantial methodological limitations constrain clinical translation. The majority of included studies were small (most n < 100), retrospective, single-centre analyses with no external validation, a pattern consistent with the broader radiomics literature [14,15]. Extreme AUC values in very small cohorts, specifically AUC 1.00 by both Aujay et al. (*n* = 22, LOOCV) [50] and Mansouri et al. (*n* = 17) [40], almost certainly reflect overfitting rather than true predictive performance. Methodological heterogeneity was pervasive across tumour types, imaging protocols, segmentation approaches, feature extraction software, machine learning algorithms, and outcome definitions, precluding meta-analytic synthesis. Publication and small-study bias could not be assessed formally without a meta-analysis, and the predominance of small studies reporting high apparent AUCs makes selective reporting likely. Segmentation reproducibility was assessed in only six studies (for example Marinelli et al., ICC = 0.83) [42] representing a significant methodological gap given the well-established sensitivity of radiomic features to segmentation variability. The adoption of automated segmentation (nnU-Net in Ocal et al.) [33] and standardised feature extraction pipelines (PyRadiomics, used in eight studies) represents progress, but adoption of the Imaging Biomarker Standardisation Initiative framework [53] remains far from routine across the included studies. Finally, the absence of biological validation linking radiomic phenotypes to underlying tumour pathophysiology limits mechanistic interpretability and the prospect of regulatory approval.

Compared with Mirza-Aghazadeh-Attari et al. [18], which identified 15 studies and performed a meta-analysis of AUC values, the current update includes 9 studies that were not in that review. The expanded and more heterogeneous evidence base identified here reinforces that quantitative pooling remains premature, and that a structured narrative synthesis is the most appropriate analytical approach at this stage of the field’s development. That review searched until 1 September 2023. Eight of the nine studies not included in it were published between 2024 and 2026 and one (Reiner et al. [35]) in 2016; the recent studies reflect the accelerating pace of radiomic research in interventional oncology and the growing interest in this area among interventional radiology and nuclear medicine groups internationally.

This review has limitations. Two cohorts overlap, because Stocker et al. state that 73 patients were reported in earlier studies including Marinelli et al., so up to 73 patients may be counted twice. Taking the highest reported AUC per study favours optimism, and the validation strata contain few studies. Scoring with the RQS and PROBAST involves judgement, although each study was scored independently by two reviewers. Only English-language studies were included.

### Future Directions

Several priority areas emerge from this synthesis. Prospective multi-centre studies with pre-specified outcome definitions, standardised imaging protocols, and rigorous external validation are most urgently needed. Harmonisation of radiomics workflows, including standardised feature extraction software, image normalisation, voxel resampling, and segmentation protocols, is essential to enable cross-study comparisons and eventual meta-analysis. The Imaging Biomarker Standardisation Initiative provides an established framework for feature standardisation that should be universally adopted in future TARE radiomics studies [53]. Similarly, reporting quality should be assessed using validated tools such as METRICS [54] to facilitate quality appraisal across future studies.

Dosiomics merits dedicated investigation in adequately powered cohorts. The combination of spatial dosimetric information with radiomic tumour characterisation is mechanistically grounded in the biology of TARE, where microsphere distribution is governed by tumour vascularity, itself a key determinant of radiomic texture. Prospective studies pairing pre-procedural radiomic assessment with post-procedural dosimetric verification could provide a comprehensive imaging-based framework integrating TARE planning, delivery, and response assessment.

The identification of biologically interpretable radiomic features linked to underlying tumour pathophysiology will be essential to facilitate mechanistic understanding and clinician adoption. Radiogenomic integration, linking radiomic phenotypes to molecular profiles, represents a particularly promising avenue in HCC, where genomic heterogeneity is well characterised and may drive differential TARE response [16]. Finally, studies demonstrating the incremental clinical value of radiomics over current standard of care, rather than standalone discriminative performance, are needed to define clinical utility. Decision curve analyses and prospective clinical impact studies examining whether radiomics improves patient selection beyond BCLC staging, Child–Pugh score, AFP, and ^99m^Tc-MAA SPECT dosimetry would represent a genuine advance toward personalised locoregional therapy for hepatic malignancies.

## 5. Conclusions

Radiomics applied to pre- and post-procedural imaging in TARE shows moderate to high apparent discrimination across hepatic tumour types and imaging modalities, but all 21 studies were at high risk of bias, none used external validation, and the median RQS was 2 of 36. Adding clinical variables to radiomic features gave only small gains. The evidence base consists of small, retrospective, mostly single-centre studies. Radiomic analyses of trial-derived cohorts (SIRFLOX, *n* = 491; SORAMIC, *n* = 297) and emerging dosiomics work are of interest but remain unvalidated. Prospective, multi-centre validation studies with standardised radiomics pipelines are required before radiomics can be routinely adopted in personalised TARE patient selection and treatment optimisation.

## Figures and Tables

**Table 1:** Methodological characteristics of included studies (*n* = 21).

First Author	Year	Journal	Tumour Type	N (Patients)	Design	Imaging Modality	Timing	Primary Outcome	Validation of Reported Estimate
Aujay [50]	2022	*Diagn Interv Imaging*	HCC	22	Retro., single-centre	MRI (CE arterial and portal)	Pre + early post	Response, mRECIST 6 mo	LOOCV
Ballı [48]	2024	*Diagn Interv Radiol*	iCC	36	Retro., single-centre	MRI (T2W, T2W FS, T1W CE equilibrium)	Pre-TARE	Response, mRECIST 6 mo	None
Blanc-Durand [44]	2018	*Oncotarget*	HCC	47	Retro., single-centre	^18^F-FDG PET/CT (whole liver)	Pre-TARE	OS and PFS	None
Gensure [43]	2012	*Acad Radiol*	Mixed	30	Retro., single-centre	Triphasic CT	Pre-TARE	Serologic response and survival	LOOCV
Ince [45]	2023	*J Vasc Interv Radiol*	HCC	82	Retro., single-centre	CE-T1W MRI	Pre-TARE	Response, EASL 3 mo	Nested 5-fold CV
Kobe [36]	2021	*Eur J Radiol Open*	Liver metastases (mixed primaries)	36 (104 lesions)	Retro., single-centre	CBCT (peri-procedural)	Pre-TARE	Lesion response, RECIST 1.1 6 mo	Hold-out (21 of 104 lesions)
Mahmoud [38]	2024	*Mol Imaging Radionucl Ther*	HCC	36	Retro. (not explicitly stated), single-centre	MRI (T2W)	Post-TARE (treatment day)	Response, mRECIST 9 mo	None
Mansouri [40]	2025	*Mol Imaging Biol*	HCC	17	Retro., single-centre	^99m^Tc-MAA SPECT/CT + ^90^Y SPECT/CT	Pre + post	Response, mRECIST 3 mo	Nested 3-fold CV
Marinelli [42]	2023	*J Vasc Interv Radiol*	HCC	76	Retro., single-centre	MRI (CE-T1W AP + ADC)	Baseline + early post (1 to 2 mo)	Complete response, mRECIST	Hold-out (*n* = 30 of 76)
Mosconi [32]	2020	*Eur Radiol*	iCC	55 (53 evaluable)	Retro., multi-centre	CT (quadriphasic)	Pre-TARE	OR, PFS, OS (RECIST 1.1)	None
Ocal [33]	2026	*Eur J Radiol Open*	CLM	491 (252 SIRT)	Post hoc analysis of SIRFLOX RCT	CT (portal venous phase)	Pre-TARE (baseline)	OS; SIRT treatment benefit	None
Reimer [37]	2018	*Cardiovasc Intervent Radiol*	Liver metastases (mixed primaries)	37	Retro., single-centre	MRI (DCE + hepatocyte-specific)	Post-TARE (mean 2.2 days)	Progressive disease, RECIST 1.1	None
Reiner [35]	2016	*Cardiovasc Intervent Radiol*	HCC	16	Retro., single-centre	CT perfusion	Pre-TARE	Response, mRECIST	None
Roll [51]	2024	*Cardiovasc Intervent Radiol*	CLM	51	Retro., single-centre	CT (portal venous, pre-TARE)	Pre-TARE	Response, RECIST 1.1 3 mo; OS	None (model fitted on validation set)
Saalfeld [34]	2023	*J Cachexia Sarcopenia Muscle*	HCC	297 (150 SIRT)	Post hoc analysis of SORAMIC RCT	CT (L3 body composition)	Pre-TARE	1-year survival	Hold-out (one-third)
Sarioglu [46]	2025	*Niger J Clin Pract*	HCC	49	Retro., single-centre	MRI (CE-T1W, T2W)	Pre-TARE	Treatment response	5-fold CV
Karahan Şen [52]	2022	*Nucl Med Commun*	HCC	33	Retro., single-centre	^18^F-FDG PET/CT	Pre-TARE	Complete versus non-complete response	None
Sozutok [47]	2024	*Rev Assoc Med Bras*	HCC	65	Retro., single-centre	MRI (T1W portal, T2W)	Pre-TARE	Complete versus non-complete response	None
Stocker [41]	2025	*Abdom Radiol*	HCC	154 (168 lesions)	Retro., single-centre	CE-MRI (T1W portal venous)	Pre-TARE	Response, mRECIST 6 mo	Repeated 80:20 CV (× 100)
Topcuoglu [49]	2026	*Jpn J Radiol*	CLM	74	Retro., single-centre	^18^F-FDG PET/CT	Pre-TARE	Local response, PERCIST 3 mo	Hold-out (*n* = 30 of 74)
Wei [39]	2020	*EJNMMI Phys*	Mixed (HCC + metastases)	30 (36 treatments, 105 lesions)	Retro., single-centre	^90^Y PET/CT	Post-TARE	Lesion response and time to progression	Nested CV

HCC = hepatocellular carcinoma; iCC = intrahepatic cholangiocarcinoma; CLM = colorectal cancer liver metastases; mRECIST = modified RECIST; PERCIST = PET Response Criteria in Solid Tumors; EASL = European Association for the Study of the Liver; OR = objective response; OS = overall survival; PFS = progression-free survival; LOOCV = leave-one-out cross-validation; CV = cross-validation; CBCT = cone-beam CT; CE = contrast-enhanced; DCE = dynamic contrast-enhanced; AP = arterial phase; ADC = apparent diffusion coefficient; FS = fat suppression; RCT = randomised controlled trial; Retro. = retrospective. N is the number of patients; lesion or treatment counts are in parentheses. For Ocal et al. and Saalfeld et al. the number of patients who received SIRT is shown in parentheses; the remainder were comparator-arm patients who did not receive TARE. Stocker et al. state that 73 and 78 patients of their cohort were reported in earlier publications, one of which is Marinelli et al.; the two cohorts therefore overlap by up to 73 patients. All 21 studies were retrospective analyses, including the two post hoc analyses of prospectively collected trial data. No study used an external validation cohort. Cross-validation alone was not counted as validation in the RQS.

**Table 2:** Radiomics workflow characteristics across included studies.

First Author	Year	Segmentation	Features (n Initial → Final)	Software	ML Algorithm
Aujay [50]	2022	Manual (ITK-SNAP), one radiologist	107 per time point → NR	PyRadiomics	Univariable L1-penalised LR (LOOCV)
Ballı [48]	2024	Manual, two radiologists (Olea Sphere)	108 per sequence → 108/4/8	Olea Sphere v3 SP2	LASSO LR rad-score + clinical LR
Blanc-Durand [44]	2018	Semi-automated whole-liver VOI (CT-derived)	108 → 39 → LASSO	CGITA	LASSO Cox (pPET-RadScore)
Gensure [43]	2012	Semi-automated (interactive contouring)	100 and 40 signature dimensions → 4 to 5	Custom (MIPAV, Iso2mesh)	SVM (LOOCV); PCA, ISOMAP, LLE
Ince [45]	2023	Manual, two radiologists (3D Slicer)	1128 → 12 (8 radiomic + 4 clinical)	3D Slicer + PyRadiomics	SVM, LR, RF, LightGBM (nested 5-fold CV)
Karahan Şen [52]	2022	Tumour VOI, 40% SUVmax threshold	44 texture + SUV parameters → 3	LIFEx	Multivariable LR
Kobe [36]	2021	Manual, two readers	104 → 15	3D Slicer + PyRadiomics	MLP ANN (10-fold CV; 21-lesion hold-out)
Mahmoud [38]	2024	Semi-automatic, single reader	87 (86 analysed) → 2	LIFEx	Univariable analysis only
Mansouri [40]	2025	Manual tumour on diagnostic images; DL whole liver	321 radiomic + 321 dosiomic + 264 DVC	PyRadiomics 3.1.0	5 selection methods x 8 ML algorithms (nested 3-fold CV)
Marinelli [42]	2023	Semi-automated (3D Slicer GrowCut), two observers	58 → 41 → 5	PyRadiomics	XGBoost ranking + LR
Mosconi [32]	2020	Semi-automated VOIs, two radiologists	24 candidate texture features → 5	LIFEx + ComBat	LASSO LR (OR); Cox (PFS, OS)
Ocal [33]	2026	Automated (nnU-Net)	100 → 20 (LASSO)	PyRadiomics 3.0.1	LASSO Cox
Reimer [37]	2018	Manual 2D ROI, largest slice	6 histogram parameters x 4 sequences	mint Lesion	Logistic regression, ROC
Reiner [35]	2016	Whole-tumour VOI, two readers	7 histogram parameters (6 analysed)	Custom	ROC analysis
Roll [51]	2024	Manual, two blinded radiologists (3D Slicer)	378 (after CCC filter) → 2	3D Slicer	Boruta + multivariable LR
Saalfeld [34]	2023	Semi-automatic (ImageJ, version not reported), L3 muscle and fat	881 per tissue → 292 → 5 to 25	PyRadiomics + MATLAB R2021a	11 ML classifiers x 9 ranking methods (990 models)
Sarioglu [46]	2025	Semi-automatic volumetric, one radiologist (LIFEx)	175 → 9 (T2W) and 12 (CE-T1)	LIFEx + MATLAB	Bagged trees, SVM (5-fold CV)
Sozutok [47]	2024	Manual, two radiologists (Olea Sphere)	108 per sequence → 5 (T1W) and 6 (T2W)	Olea Sphere v3 SP2	LASSO LR rad-score + clinical LR
Stocker [41]	2025	Manual VOI, main reader (ICC on subsets)	45 radiomic + clinical (71 candidates)	LIFEx v5.1	Elastic-net LR (repeated 80:20 CV)
Topcuoglu [49]	2026	SUVbw > 4 threshold with manual correction (3D Slicer)	107 → 5	3D Slicer + PyRadiomics	RF, XGBoost, LR
Wei [39]	2020	Radiologist contours on diagnostic CT/MR registered to PET	46 → 15 robust → Bo-LASSO	In-house MATLAB toolbox (IBSI-benchmarked)	Bootstrapped LASSO LR + Cox (nested CV)

NR = not reported; LR = logistic regression; SVM = support vector machine; RF = random forest; MLP ANN = multilayer perceptron artificial neural network; LASSO = least absolute shrinkage and selection operator; CCC = concordance correlation coefficient; ICC = intraclass correlation coefficient; VOI = volume of interest; ROI = region of interest; DVC = dose-volume constraint; DL = deep learning; IBSI = Imaging Biomarker Standardisation Initiative; CV = cross-validation; LOOCV = leave-one-out cross-validation. PyRadiomics was used in eight studies (Aujay, Ince, Kobe, Marinelli, Mansouri, Ocal, Saalfeld, Topcuoglu). Wei et al. used an in-house MATLAB toolbox benchmarked against IBSI and not PyRadiomics.

## Data Availability

No new data were created or analysed in this study. Data sharing is not applicable to this article.

## Supplementary Materials

The following supporting information can be downloaded at: https://www.mdpi.com/article/10.3390/diagnostics16203322/s1, Supplementary Material S1: Full search strategies for each database; Supplementary Material S2: Radiomics Quality Score scoring conventions and per-study scores; Supplementary Material S3: PROBAST risk of bias and applicability assessments. References [15,24,32,33,34,35,36,37,38,39,40,41,42,43,44,45,46,47,48,49,50,51,52] are cited in the Supplementary Materials.
