# Supplementary material for: Radiomics-Based Outcome Prediction in Transarterial Radioembolization (TARE) of Hepatic Malignancies: A Systematic Review and Quality Appraisal
Source: Diagnostics (Basel). 2026 Oct 13;16(20):3322. doi: 10.3390/diagnostics16203322 (PMC13703477; doi:10.3390/diagnostics16203322)
Supplement: Supplementary file 1 [file diagnostics-16-03322-s001.zip › diagnostics-4526831-supplementary materials-1013.pdf]

Supplementary Material

Reference numbers in square brackets refer to the reference list of the main article.

Supplementary Material S1: Full Search Strin

The following search strings were applied in each database with no date restrictions. The final searches were conducted on 28/05/2026.

PubMed (via Medline)

("radioembolization"[tiab] OR "radio-embolization"[tiab] OR "TARE"[tiab] OR "SIRT"[tiab] OR "selective internal radiation"[tiab] OR "yttrium-90"[tiab] OR "Y-90"[tiab] OR "Y90"[tiab] OR "90Y"[tiab] OR "microsphere"[tiab]) AND ("radiomics"[tiab] OR "texture analysis"[tiab] OR "texture feature"[tiab] OR "machine learning"[tiab] OR "deep learning"[tiab] OR "artificial intelligence"[tiab] OR "quantitative imaging"[tiab] OR "dosiomics"[tiab] OR "radiomic"[tiab])

Embase

('radioembolization' OR 'radio-embolization' OR 'TARE' OR 'SIRT' OR 'selective internal radiation therapy' OR 'yttrium-90' OR 'Y-90' OR 'Y90' OR '90Y' OR 'microsphere') AND ('radiomics' OR 'radiomic' OR 'texture analysis' OR 'texture feature' OR 'machine learning' OR 'deep learning' OR 'artificial intelligence' OR 'quantitative imaging' OR 'dosiomics')

Scopus

TITLE-ABS-KEY(("radioembolization" OR "radio-embolization" OR "TARE" OR "SIRT" OR "selective internal radiation" OR "yttrium-90" OR "Y-90" OR "Y90" OR "90Y" OR "microsphere") AND ("radiomics" OR "radiomic" OR "texture analysis" OR "texture feature" OR "machine learning" OR "deep learning" OR "artificial intelligence" OR "quantitative imaging" OR "dosiomics"))

Web of Science

TS=("radioembolization" OR "radio-embolization" OR "TARE" OR "SIRT" OR "selective internal radiation" OR "yttrium-90" OR "Y-90" OR "Y90" OR "90Y" OR "microsphere") AND TS=("radiomics" OR "radiomic" OR "texture analysis" OR "texture feature" OR "machine learning" OR "deep learning" OR "artificial intelligence" OR "quantitative imaging" OR "dosiomics")

ClinicalTrials.gov

Condition or disease: "liver neoplasms" OR "hepatocellular carcinoma" OR "cholangiocarcinoma" OR "liver metastases" OR "hepatic metastases". Intervention or treatment: radioembolization OR radioembolisation OR "yttrium-90" OR "Y-90" OR Y90 OR SIRT OR TARE OR "selective internal radiation therapy". Other terms: radiomics OR "texture analysis" OR "machine learning" OR "artificial intelligence".

Supplementary Material S2: Radiomics Quality Score (RQS) assessment

The RQS is a 16-item tool with a possible total score ranging from -8 to 36 [15]. Each study was scored independently by two reviewers (RG and HT), with disagreements resolved by a final decision of the senior author (MK). Where the published tool leaves room for interpretation, the following conventions were applied. Cross-validation alone was scored as no validation (-5), and a random hold-out or split of the same cohort as validation from the same institute (+2), provided the held-out data were not used to fit or select the model (Roll et al. [51] fitted their logistic model on the validation set and were scored -5). Discrimination statistics scored a point only when an AUC or C-index was reported with a confidence interval or p value, and the resampling point only when the reported estimate came from cross-validation or bootstrapping. Feature reduction scored +3 where dimensionality reduction or multiplicity adjustment was performed and -3 where neither was done. Comparison with a gold standard scored +2 only against an established standard (for example mRECIST, size criteria or BCLC stage), and an ad hoc clinical comparator model scored 0. Combining clinical or dosimetric variables with radiomic features scored under multivariable analysis with non-radiomic features. Post hoc analyses of trial data were not scored as prospective. Biological correlation required correlation with histology, genomics or another biological reference and was not credited for speculative discussion.

Study	1	2	3	4	5	6	7	8	9	10	11	12	13	14	15	16	Total
Aujay [50]	1	0	0	1	3	0	0	1	2	0	0	-5	2	0	0	0	**5**
Ballı [48]	1	0	0	0	3	1	0	1	1	0	0	-5	0	0	0	0	**2**
Blanc-Durand [44]	1	0	0	0	3	1	0	1	0	0	0	-5	0	0	0	0	**1**
Gensure [43]	1	0	0	0	3	0	0	0	1	0	0	-5	0	0	0	0	**0**
Ince [45]	1	1	0	0	3	1	0	0	2	0	0	-5	0	0	0	1	**4**
Kobe [36]	1	1	0	0	3	0	0	0	1	0	0	2	0	0	0	0	**8**
Mahmoud [38]	1	0	0	0	-3	0	0	1	1	0	0	-5	0	0	0	0	**-5**
Mansouri [40]	1	0	0	1	3	1	0	0	2	0	0	-5	0	0	0	0	**3**
Marinelli [42]	1	1	0	1	3	0	0	1	1	0	0	2	2	0	0	0	**12**
Mosconi [32]	1	0	0	0	3	1	0	1	1	0	0	-5	0	0	0	0	**2**
Ocal [33]	1	0	0	0	3	1	0	1	0	0	0	-5	0	0	0	0	**1**
Reimer [37]	1	0	0	0	-3	0	0	1	1	0	0	-5	0	0	0	0	**-5**
Reiner [35]	1	1	0	0	-3	0	0	1	0	0	0	-5	0	0	0	0	**-5**
Roll [51]	0	1	0	0	3	0	0	1	1	0	0	-5	0	0	0	0	**1**
Saalfeld [34]	0	0	0	0	3	0	0	0	1	0	0	2	0	0	0	0	**6**
Sarioglu [46]	1	0	0	0	3	1	0	1	2	0	0	-5	0	0	0	0	**3**
Karahan Şen [52]	1	0	0	0	3	1	0	1	1	0	0	-5	0	0	0	0	**2**
Sozutok [47]	1	0	0	0	3	1	0	1	1	0	0	-5	0	0	0	0	**2**
Stocker [41]	1	1	0	0	3	1	0	1	2	0	0	-5	0	0	0	0	**4**
Topcuoglu [49]	1	0	0	0	3	1	0	1	1	0	0	2	0	0	0	0	**9**
Wei [39]	1	0	1	0	3	1	0	0	2	2	0	-5	0	0	0	1	**6**

Item key: 1. Image protocol (max 2); 2. Multiple segmentations (max 1); 3. Phantom study (max 1); 4. Multiple time points (max 1); 5. Feature reduction (max 3); 6. Non-radiomic features (max 1); 7. Biological correlates (max 1); 8. Cut-off analysis (max 1); 9. Discrimination (max 2); 10. Calibration (max 2); 11. Prospective study (max 7); 12. Validation (max 5); 13. Gold standard (max 2); 14. Clinical utility (max 2); 15. Cost-effectiveness (max 1); 16. Open science (max 4). Item 1 combines protocol quality (+1) and public protocol (+1); item 9 combines discrimination (+1) and resampling (+1); item 10 combines calibration (+1) and resampling (+1). Item

5 ranges from -3 to +3 and item 12 from -5 to +5. Total scores (maximum 36): median 2 (interquartile range 1 to 5), range -5 to 12; the highest score, 12 (Marinelli et al. [42]), is 33% of the maximum. Studies with a negative total are shaded.

Supplementary Material S3: PROBAST assessment of risk of bias and applicability

Risk of bias and applicability were assessed with PROBAST [24], which assesses risk of bias in four domains (participants, predictors, outcome and analysis) and applicability concerns in three (participants, predictors and outcome). Each study was assessed independently by two reviewers (RG and HT), with disagreements resolved by a final decision of the senior author (MK). A domain was rated high if any signalling question indicated a high risk of bias, low if all signalling questions were answered favourably, and unclear otherwise. The overall risk of bias was rated low only if all four domains were low and high if any domain was high. In the analysis domain, risk was judged high where the number of outcome events per candidate predictor was far below 10, where univariable screening or feature selection used the whole cohort before validation, where no calibration was reported, or where there was no validation other than cross-validation or a very small hold-out set. Predictors extracted from post-treatment imaging were not judged an applicability concern because the review question includes post-procedural models.

Study	Participants	Predictors	Outcome	Analysis	Overall risk of bias	Applicability: participants	Applicability: predictors	Applicability: outcome	Overall applicability	Principal reason for high risk in the analysis domain
Aujay [50]	High	Unclear	Low	High	High	Low	Low	Low	Low	22 patients (8 responders) vs 107 x 3 candidate features; univariable selection; LOOCV only; AUC 1.00; no calibration; no external validation
Ballı [48]	High	Low	Low	High	High	Low	Low	Low	Low	13 responders vs 108 candidate features per phase; no validation; apparent AUC; no calibration; DeLong on apparent AUCs
Blanc-Durand [44]	Low	Low	Low	High	High	Low	Low	Low	Low	33 deaths / 30 progressions vs 39 candidate features after filtering; X-tile data-driven dichotomisation; no validation; no calibration; no discrimination statistic
Gensure [43]	Unclear	Unclear	High	High	High	Low	Low	Low	Low	n=30 (13 serologic responders); 100-d and 40-d signatures; dimension chosen on same LOOCV used for evaluation; no external validation; no calibration

Study	Participants	Predictors	Outcome	Analysis	Overall risk of bias	Applicability: participants	Applicability: predictors	Applicability: outcome	Overall applicability	Principal reason for high risk in the analysis domain
Ince [45]	Unclear	Low	Low	High	High	Low	Low	Low	Low	17 non-responders vs 1128 candidate features (12 final); SMOTE/undersampling before CV; feature selection on full data; CIs reflect CV repetition; no calibration; no external validation
Kobe [36]	High	Unclear	Low	High	High	Low	Low	Low	Low	104 lesions from 36 patients analysed as independent (clustering ignored); random lesion-level split (83/21) so same-patient lesions can span train and test; 19 PD lesions only; 104 candidate features; AUC without CI; no calibration
Mahmoud [38]	Unclear	Unclear	Unclear	High	High	Low	Low	Low	Low	8 non-responders vs 86 candidate features; univariable Mann–Whitney without multiplicity control; the two features quoted (AUC 0.71) were not significant (p=0.07); no validation; no calibration
Mansouri [40]	Unclear	Low	Low	High	High	Low	Low	Low	Low	5 responders vs 12 non-responders; 906 candidate features (321 radiomic, 321 dosiomic, 264 DVC) in 1,440 model combinations with best model reported (AUC 1.00); no feature reached FDR significance; SMOTE; no calibration; no external validation
Marinelli [42]	Low	Low	Low	High	High	Low	Low	Low	Low	16 patients without CR (60 CR) vs 58 candidate features; 30-patient hold-out with wide CI (0.77-1.00); DeLong p=0.12 and 0.15 versus comparators; missing ADC imputed; no calibration; not externally validated

Study	Participants	Predictors	Outcome	Analysis	Overall risk of bias	Applicability: participants	Applicability: predictors	Applicability: outcome	Overall applicability	Principal reason for high risk in the analysis domain
Mosconi [32]	Low	Unclear	Unclear	High	High	Low	Low	Unclear	Unclear	23 non-responders versus 24 candidate features (3 phases x 8) with p-value pre-screening; no independent or hold-out validation; apparent AUC only; no calibration
Ocal [33]	Low	Low	Low	High	High	High	Low	Low	High	No discrimination or calibration reported; no internal or external validation; 100 radiomic features plus clinical variables and treatment interactions with LASSO (no confidence intervals); risk groups dichotomised at 50% predicted probability
Reimer [37]	High	High	Unclear	High	High	Low	Unclear	Low	Unclear	13 non-progressors versus 24 candidate parameter tests without correction; cut-offs derived and assessed in same data; no validation; no calibration
Reiner [35]	High	Unclear	Low	High	High	Low	Low	Low	Low	8 responders and 8 non-responders with seven candidate parameters; cut-off chosen for high sensitivity on the same data; no validation; no calibration
Roll [51]	Unclear	Low	Unclear	High	High	Low	Low	Low	Low	24 responders versus 27 non-responders with 378 candidate features; logistic model fitted on the same 30% validation set on which the AUC is reported (0.75, CI 0.48-1.00, about 15 patients); no calibration; no external validation

Study	Participants	Predictors	Outcome	Analysis	Overall risk of bias	Applicability: participants	Applicability: predictors	Applicability: outcome	Overall applicability	Principal reason for high risk in the analysis domain
Saalfeld [34]	High	Unclear	Low	High	High	High	Low	Low	High	990 models (9 selectors x 10 sets x 11 classifiers) compared; test sets of about 50 patients per arm; single split; no calibration; accuracy-based selection; no external validation
Sarioglu [46]	Unclear	Low	Unclear	High	High	Low	Low	Low	Low	11 non-responders versus 175 candidate features; feature selection outside CV loop; several classifiers chosen on accuracy; CV only; no calibration; reported accuracy, sensitivity and specificity cannot be reconciled with a 38:11 class split under one positive-class definition
Karahan Şen [52]	Unclear	Unclear	Unclear	High	High	Low	Low	Low	Low	14 complete responders versus 44 texture features plus conventional SUV metrics with p-value filtering; cut-off derived on same data; no validation; no calibration
Sozutok [47]	Unclear	Unclear	Low	High	High	Low	Low	Low	Low	36 non-complete responders versus 108 candidate features per sequence; rad-scores built and assessed on the same 65 patients; backward stepwise clinical selection; no validation; no calibration; discussion cites AUC 0.88 and an OS analysis not reported in Results
Stocker [41]	Unclear	Unclear	Unclear	High	High	Low	Low	Low	Low	55 non-response lesions versus 45 radiomic plus 26 clinical candidates (about 0.8 events per variable); random lesion-level 80:20 splits allow patient leakage; CV only; no calibration; no external validation

Study	Participants	Predictors	Outcome	Analysis	Overall risk of bias	Applicability: participants	Applicability: predictors	Applicability: outcome	Overall applicability	Principal reason for high risk in the analysis domain
Topcuoglu [49]	Unclear	Low	Unclear	High	High	Low	Low	Low	Low	30-patient test set with CI reaching 0.5; 107+16 candidate predictors for 24 events; no calibration; no external validation; missing laboratory values median-imputed
Wei [39]	Unclear	Unclear	Low	High	High	Low	Low	Low	Low	Lesion-level analysis with non-independent lesions; 14 progression events for 17 candidate variables; ADASYN oversampling; nested CV only; no external validation

Overall risk of bias was high for all 21 studies, and the analysis domain was rated high in every study. Applicability was rated a high concern for Ocal et al. [33] and Saalfeld et al. [34], because 239 of 491 and 147 of 297 patients respectively were comparator-arm patients who did not receive TARE, and unclear for the predictors domain in Reimer et al. [37] (post-treatment MRI with the time point chosen using the outcome) and the outcome domain in Mosconi et al. [32] (best response at any follow-up). All other applicability ratings were low.
